# Malignant triton tumor with thoracic region as the initial presentation: a case report

**DOI:** 10.3389/fonc.2026.1764543

**Published:** 2026-03-27

**Authors:** Hang Yin, Xindi Zhang, Zhan Huang, Xiaotian Zhang, Qian Li, Tao Hu, Xiaoliang Shi, Changbin Zhu, Huabing Wei

**Affiliations:** 1 Department of Thoracic Surgery, Renji Hospital, School of Medicine, Shanghai Jiaotong University, Shanghai, China; 2 Department of Translational Medicine, Amoy Diagnostics Co., Ltd, Xiamen, China

**Keywords:** case report, immunotherapy, malignant triton tumor, neurofibromatosis type 1, single-cell sequencing, thoracic tumor

## Abstract

**Background:**

Malignant Triton Tumor (MTT) is a rare, highly aggressive peripheral nerve sheath tumor characterized by rhabdomyoblastic differentiation, seldom occurring primarily in the thoracic cavity. This report presents a rare case of MTT initially manifesting as chest pain, and reviews relevant literature to summarize its clinical features and therapeutic strategies.

**Methods:**

A 23-year-old woman experienced chest pain for ten days and her condition worsened within 3 days. The patient had a known history of neurofibromatosis type 1 (NF1) and exhibited classic café-au-lait spots on physical examination. Diagnosis was confirmed via chest CTA, PET-CT, bronchoscopy, ultrasound, and histopathology. After surgical resection, the tumor recurred rapidly, prompting multiple treatments including chemotherapy, targeted therapy, and combination immunotherapy.

**Results:**

Pathology after right lower lobectomy and chest wall resection confirmed MTT (S-100+/Desmin+/Myogenin+). Recurrence in the mediastinum was detected two months postoperatively. Disease stabilization was achieved using cadonilimab, apatinib, and ifosfamide/etoposide. Notably, the first application of single-cell RNA sequencing (scRNA-seq) in MTT, combined with the CopyKAT algorithm, distinguished malignant from non-malignant cells and revealed a heterogeneous tumor microenvironment composed of distinct functional cell populations, highlighting the tumor’s high degree of heterogeneity.

**Conclusion:**

This case underscores the importance of considering MTT in young NF1 patients presenting with intrathoracic masses. An individualized, multimodal treatment approach may extend survival. scRNA-seq provides valuable insights into the molecular landscape of MTT and hold promise for guiding precision therapy in the future.

## Introduction

Malignant Triton Tumor (MTT) is a rare and highly aggressive subtype of malignant peripheral nerve sheath tumor (MPNST) (1, 2). Epidemiological data indicate that MTT accounts for approximately 1%–3% of all MPNSTs, with the overall incidence of MPNST estimated at 0.8–1.5 per million population (3). Histologically, MTT is characterized by a spindle cell sarcoma of Schwann cell origin with rhabdomyoblastic differentiation. Approximately 65%–75% of cases are associated with Neurofibromatosis Type 1 (NF1) (4). Due to its dual neurogenic and rhabdomyoblastic differentiation, MTT exhibits highly aggressive biological behavior, often presenting with rapid local invasion, a high postoperative recurrence rate (> 50%), and a significant risk of metastasis, particularly to the lungs (30%–45%). The 5-year overall survival rate is reported to be less than 20% (5).

MTT most commonly arises from peripheral nerves of the trunk and extremities (accounting for 75%–80% of cases), while primary intrathoracic tumors are rare, representing only 5%–10% of cases (6). When MTT presents primarily in the chest, its non-specific symptoms-such as chest pain or dyspnea—can easily lead to misdiagnosis as pleural mesothelioma or a mediastinal neurogenic tumor. Accurate diagnosis relies heavily on histopathological and immunohistochemical examination (7). Pathological diagnosis of MTT is particularly challenging: it must be differentiated from other spindle cell sarcomas (such as synovial sarcoma and fibrosarcoma), as well as metastatic rhabdomyosarcoma. Immunohistochemical evidence of rhabdomyoblastic differentiation (e.g., Desmin and Myogenin positivity) is essential for diagnosis, but in some cases this component may be focal and easily overlooked, increasing the risk of misdiagnosis (8).

Currently, wide surgical excision remains the only potentially curative treatment. However, achieving R0 resection in intrathoracic MTT is often difficult due to proximity to major blood vessels or the spine, and the efficacy of adjuvant radiotherapy and chemotherapy remains controversial (9, 10).

Herein, we report the case of a young female patient who presented with chest pain as the initial symptom of MTT. The primary thoracic tumor measured over 15 cm in diameter and was associated with NF1-related skin manifestations and early postoperative recurrence. Through an integrated approach combining imaging, histopathology, and single-cell RNA sequencing (scRNA-seq), this report explores the diagnostic challenges, molecular characteristics, and therapeutic difficulties associated with MTT. Our goal is to raise clinical awareness of this rare tumor and provide a reference for the development of individualized treatment strategies.

## Case presentation

**Figure 1:**
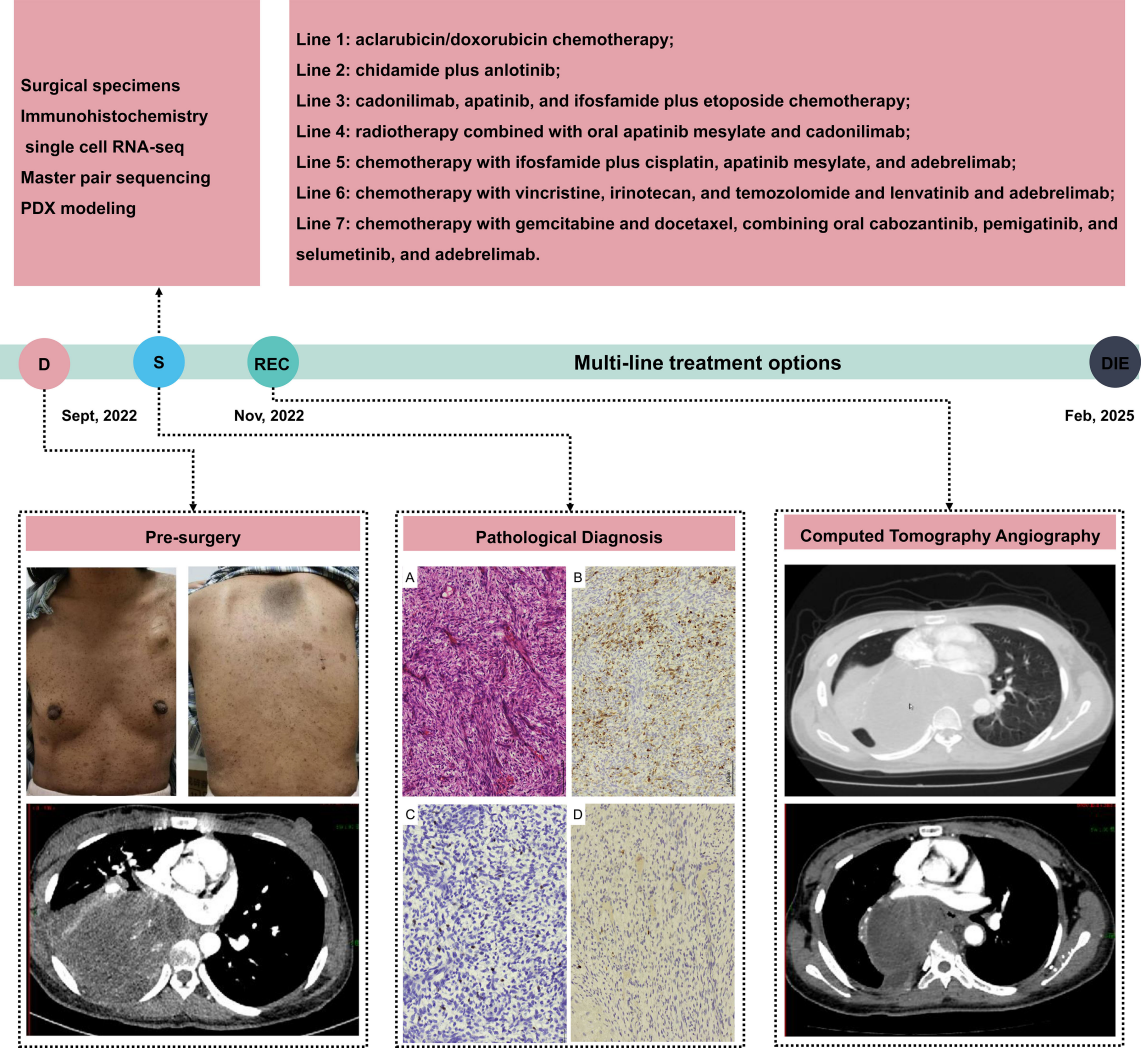
Timeline of this case’s disease from diagnosis to stable. Pre-surgery: up panel-physical examination, anterior and posterior views showing café-au-lait macules; down panel-initial CTA (axial chest CT) revealed a large mass occupying the right lower lung. Pathological diagnosis: Histopathology and Immunohistochemistry Images, **(A)** Hematoxylin and eosin staining shows densely packed spindle cells with cystic changes and rhabdomyoblastic differentiation. **(B)** Desmin positive. **(C)** Myogenin positive. **(D)** S-100 protein partially positive. Computed tomography angiography: up panel-recurrent CT image; down panel-follow-up chest CTA showing posterior mediastinal recurrence. D, diagnosis; S, Surgery; REC, Recurrence; scRNA-seg, Single-cell RNA sequencing; PDX, Patient-Derived Xenografts.

A 23-year-old woman was admitted in September 2022 for a 10-day history of chest pain that had worsened over the past three days. She had a known history of NF1. The physical examination revealed typical café-au-lait spots (**Figure 1**), placing her at high risk for developing neurogenic tumors. A chest computed tomography angiography (CTA), revealed a large mixed-density mass with slightly hypodense characteristics in the right hemithorax, measuring approximately 11 × 9 × 11 cm (**Figure 1**). The lesion showed irregular thickness at the margins and demonstrated delayed, heterogeneous enhancement following contrast administration, with more prominent enhancement along the periphery. Routine blood tests at admission, including complete blood count and basic biochemistry, were within normal limits. A mild elevation of CA-125 (45.77 U/mL) was noted. Other common tumor markers, including CEA and CA19-9, were within normal limits.

On September 21, 2022, the patient underwent a right lower lobectomy and resection of a chest wall lesion. Postoperative pathological examination confirmed a MTT—a MPNST with rhabdomyoblastic differentiation. Histological analysis revealed densely packed spindle cells. The diagnosis was further supported by immunohistochemistry, which showed that tumor cells stained positive for S-100, Desmin, and Myogenin. The Ki-67 proliferation index was high at 80% (**Figure 1**). Genetic analysis of the surgical specimen using a 571-gene panel (AmoyDx^®^ Master Panel) identified two pathogenic mutations: *NF1* (c.2547del; VAF 79.97%) and *SUZ12* (c.677del; VAF 83.96%). Furthermore, the tumor was classified as microsatellite stable (MSS) and homologous recombination deficiency-negative (HRD-negative), with a low tumor mutational burden (TMB-low) of 2.5 Muts/Mb.

Two months after surgery, a mediastinal recurrence measuring 96×68 mm was detected. The patient received two cycles of chemotherapy with aclarubicin and doxorubicin as part of a clinical trial. However, a computed tomography (CT) scan on January 16, 2023, revealed significant tumor progression, with the lesion enlarging to 147×106 mm. A patient-derived xenograft (PDX) model was then established to guide therapeutic decisions. Consequently, on January 17, 2023, the patient began targeted therapy with chidamide and anlotinib. This regimen proved ineffective; after two cycles, the tumor dimensions had changed from 147×106 mm (January 16, 2023) to 145×116 mm (February 26, 2023), indicating progressive disease. Subsequently, the patient was treated with a triple-combination regimen: cadonilimab (a bispecific PD-1/CTLA-4 antibody), apatinib (a VEGFR2 inhibitor), and ifosfamide/etoposide chemotherapy. This therapy yielded a partial response after seven cycles. The patient then proceeded with maintenance therapy, receiving an additional seven cycles of cadonilimab and apatinib. However, a subsequent efficacy assessment on December 27, 2023, revealed tumor progression, with the lesion now measuring 45×28 mm. On January 11, 2024, the patient commenced a 22-day course of radiotherapy administered concurrently with oral apatinib and cadonilimab.

However, a CT scan on February 26, 2024, revealed disease progression, with the mediastinal tumor having enlarged to 79×47 mm. Consequently, the treatment was changed to a triple-combination regimen administered every three weeks (Q3W), consisting of ifosfamide/cisplatin chemotherapy, apatinib, and the anti-PD-L1 antibody adebrelimab, for nine cycles. During this treatment course, a magnetic resonance imaging (MRI) scan on April 6, 2024, identified a new mass in the left popliteal fossa measuring 20×33×17 mm. Serial efficacy evaluations confirmed a gradual reduction in the mediastinal tumor, which measured 70×47 mm on May 15, 2024, and 68×47 mm on June 29, 2024. This outcome indicated a partial response to the multimodal regimen. Despite this partial response, a significant disease burden persisted, prompting a regimen adjustment on September 24, 2024. The patient then completed four cycles of a new combination therapy: (1) vincristine, irinotecan, and temozolomide chemotherapy; (2) oral lenvatinib; and (3) adebrelimab. As of December 4, 2024, follow-up imaging showed that while the mediastinal tumor was stable, new metastatic lesions had developed in the right lower lung lobe (32×37 mm) and the right posterior hepatic lobe (8 mm). On December 21, 2024, in response to widespread metastatic progression, the strategy was revised to an aggressive multidrug regimen: (1) gemcitabine/docetaxel chemotherapy; (2) multi-targeted therapy with cabozantinib, pemigatinib, and selumetinib; and (3) adebrelimab. Despite these intensive and continuously adjusted therapeutic efforts, the patient succumbed to the disease in February 2025.

**Figure 2:**
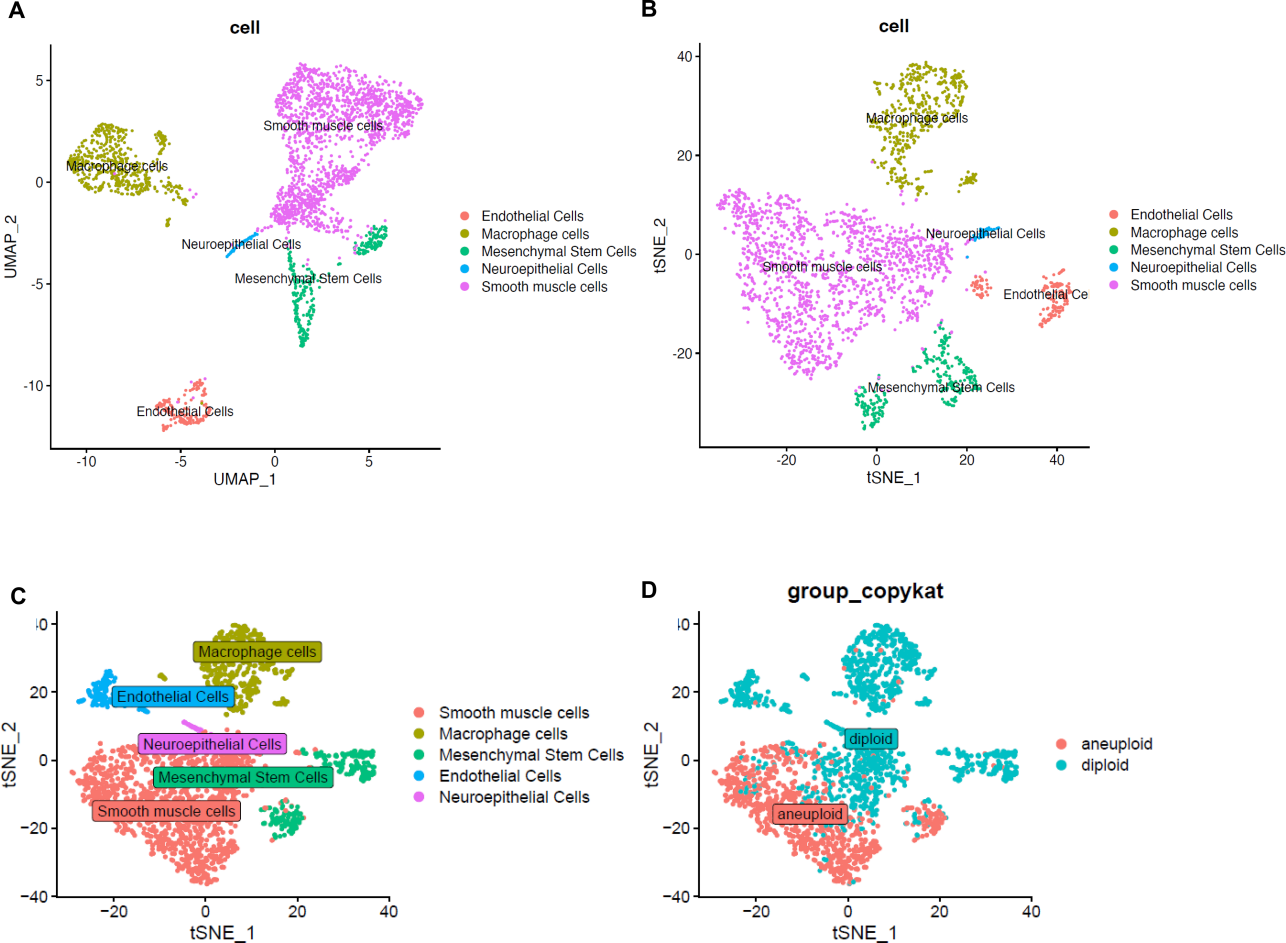
Projection of scRNA-seq data. Distribution of major cell types in MTT scRNA-seq data visualized by UMAP **(A)** and t-SNE **(B)**.​ t-SNE visualization of MTT Cells with CopyKAT classification **(C, D)**.

We performed scRNA-seq on the MTT sample from this patient. As illustrated in **Figures 2A, B**, UMAP and t-SNE dimensionality reduction revealed distinct cell cluster distributions, reflecting multiple cellular subpopulations within the tumor tissue, including smooth muscle cells, macrophages, mesenchymal stem cells, endothelial cells, and neuroepithelial cells. Using the CopyKAT algorithm, we successfully discriminated aneuploid tumor cells from diploid normal cells, thereby clarifying the composition of malignant and non-malignant compartments (**Figures 2C, D**). This result elucidated the cellular interactions between tumor components and the surrounding microenvironment.

## Discussion

We report a 23-year-old female patient with MTT and provided a systematic analysis of the tumor’s clinical features, pathological characteristics, and treatment strategies.

Notable clinical features of this case include: 1) Young age (23 years), this case demonstrates that MTT can occur in young individuals, which aligns with the findings of Brooks et al., who noted that although most MTTs occur between ages 30 and 50, the disease is not limited to older populations (11); 2) Confirmed NF1 history: characterized by typical café-au-lait spots, suggesting the tumor may arise from abnormal proliferation related to a genetic background, with approximately 70% of MTT cases associated with NF1, a statistic supported by multiple studies (12); 3) Uncommon tumor location: the tumor originated in the right thoracic cavity and invaded the posterior chest wall and bronchial structures, leading to lung compression and airway narrowing, clinically manifesting as chest pain and dyspnea. Compared to common sites such as extremities or the retroperitoneum, thoracic-origin MTTs are exceedingly rare, and only sporadic case reports available (13); 4) Imaging characteristics: radiological examination showed a large, ill-defined, and highly vascular mass with high metabolic activity indicated by PET-CT, consistent with the highly aggressive nature of the tumor. This finding is in line with prior reports describing MTTs as large, ill-defined tumors with frequent cystic degeneration, necrosis, and hemorrhage (14); 5) Rapid recurrence: despite underwent surgical resection, the patient experienced mediastinal recurrence with vertebral destruction within a short postoperative period, confirming the high recurrence rate and metastatic potential of MTT. Consistent with previous study which showed a median recurrence time of 6 months and 50% of patients experiencing local progression or recurrence (15). This unusually rapid recurrence can be attributed to both clinical and molecular factors. Clinically, the tumor exhibited a remarkably high Ki-67 proliferation index (~80%), and immediate postoperative adjuvant therapy was not administered, which may have allowed residual microscopic disease to progress rapidly. At the molecular level, our single-cell transcriptomic analysis (**Supplementary Figure 1**) uncovered specific drivers: using CopyKAT, we identified Cluster 0 as the primary aneuploid malignant population. These cells exhibited a smooth muscle-like differentiation signature (high expression of ACTA2, MYH11, TAGLN) and were significantly enriched for MYC TARGETS V1, E2F TARGETS, and mTORC1 SIGNALING pathways, providing a direct molecular explanation for the high proliferative capacity. Furthermore, the malignant cells showed strong enrichment for OXIDATIVE PHOSPHORYLATION and EPITHELIAL MESENCHYMAL TRANSITION (EMT) pathways, indicating high metabolic demand and intrinsic invasive properties that facilitated local recurrence. Beyond the tumor cells, we identified a population of Tumor-Associated Macrophages (TAMs) (Cluster 1, expressing C1QA, SPP1, APOE) with M2-like features, which are known to suppress anti-tumor immunity and promote tissue remodeling, thereby contributing to recurrence. In summary, the rapid recurrence was driven by a metabolically active, hyper-proliferative aneuploid cell population with EMT features, supported by an immunosuppressive macrophage microenvironment.

The diagnosis of MTT is based on histopathological and immunohistochemical findings. Histopathologically, it presents as a spindle cell sarcoma with rhabdomyoblastic differentiation, which is confirmed by positive immunohistochemical staining for myogenic markers such as desmin and myogenin. The primary differential diagnosis is rhabdomyosarcoma, although both tumors exhibit rhabdomyoblastic differentiation, MTT is distinguished by its schwannoma-like histological features, which are typically absent in rhabdomyosarcoma. Furthermore, they demonstrate key epidemiological differences: rhabdomyosarcoma is more prevalent in children and adolescents, whereas adult MTT is frequently associated with NF1 or a history of radiotherapy (16). MPNST with myogenic differentiation, which can be difficult to distinguish from MTT and requires comprehensive assessment of tissue origin and clinical background, with Daimaru et al. being the first to broaden the definition of MTT to include sporadic cases and emphasize its close relationship with MPNST (17). Other soft tissue sarcomas, such as liposarcoma or synovial sarcoma, which can be effectively differentiated by immunohistochemistry. In addition, imaging should help distinguish MTT from intrathoracic masses like thymoma, lymphoma, and lung cancer, especially when tumor location is atypical.

Currently, no standardized treatment guidelines for MTT exist; consequently, management strategies are largely adapted from protocols for other soft tissue sarcomas. Complete surgical resection with negative margins (R0 resection) remains the cornerstone of therapy, although achieving this is often challenging due to tumor depth and proximity to vital structures. The insufficiency of surgery as a monotherapy was highlighted in this case, as the patient developed mediastinal recurrence just two months after undergoing right lower lobectomy and chest wall resection.

Adjuvant radiotherapy and chemotherapy are widely employed in high-risk or incompletely resected cases. Radiotherapy reduces local recurrence risk, while chemotherapy may control distant metastasis. In this case, the absence of immediate postoperative adjuvant therapy may have contributed to early disease progression. Upon recurrence, various treatment approaches were attempted, this outcome suggests that in cases where conventional therapies fail, a multi-modal approach combining immunotherapy, targeted therapy, and chemotherapy may offer new hope for patients with advanced MTT and warrants further investigation. Notably, Tsimpinos et al. reported some success with the IA regimen (ifosfamide + doxorubicin) as second-line treatment (18). In contrast, our patient responded better to a triple regimen of cadonilimab (a bispecific PD-1/CTLA-4 antibody), apatinib (a VEGFR2 inhibitor), and ifosfamide/etoposide chemotherapy after IA failure, highlighting such a multimodal approach represents a promising salvage strategy for advanced, refractory MTT when standard options are exhausted.

Although usually an active multimodal treatment approach is adopted, especially in younger patients, it is necessary to carefully weigh the relationship between treatment intensity, potential survival benefits, and quality of life. For advanced or refractory cases, palliative and symptom-oriented care may become the main focus. Hashimoto et, al. recently published a case report highlighting this challenge, describing a patient with advanced metastatic MTT whose surgery was mainly aimed at relieving pain and improving mobility, which highlights the role of palliative intervention in managing severe symptoms and improving quality of life when a cure is not achievable (5). This perspective is crucial for comprehensive management, especially in such cases of poor prognosis and highly aggressive tumors.

This study presents the first application of scRNA-seq in a patient with MTT, integrating transcriptomic profiling with the CopyKAT algorithm to distinguish tumor cells from non-tumor components based on aneuploidy status. The analysis revealed that the tumor was predominantly composed of smooth muscle-like cells with aneuploidy and exhibited features of both neurogenic and myogenic differentiation, consistent with the pathological hallmark of MTT. In addition, diverse diploid stromal and immune cell populations, including macrophages, endothelial cells, mesenchymal stem cells, and neuroepithelial cells, were identified, highlighting a complex and interactive tumor microenvironment. These findings underscore the profound cellular heterogeneity of MTT and provide insights into the potential role of non-malignant cell types in supporting tumor progression. Future integration of multi-omics data may uncover novel therapeutic targets and advance personalized treatment strategies for this rare and aggressive tumor. This case is uniquely significant in three key aspects. First, it demonstrates a rare primary location: the initial thoracic presentation with bronchial compression and pleural invasion represents an uncommon phenotype. Second, it showcases successful multi-line therapy: continuous adjustment of treatment strategies achieved long-term disease control, providing valuable reference for similar cases. Third, it exemplifies integration of cutting-edge technology: scRNA-seq analysis revealed the tumor microenvironment and identified potential targets for individualized therapy. These findings echo the work of Velagaleti et al. on 9p chromosomal abnormalities in MTT (19), providing a new perspective for elucidating the molecular mechanisms of MTT and identifying novel therapeutic targets.

We acknowledge the inherent limitations of this study. Firstly, as a single-case report, our findings are descriptive and cannot be generalized to all MTT patients. The clinical course and therapeutic responses observed here are specific to this individual and may not represent the broader disease spectrum. Secondly, while the application of scRNA-seq provides unprecedented resolution into tumor heterogeneity and microenvironment, the data generated from a single sample require validation in larger, prospective cohorts to confirm the biological and clinical significance of the identified cellular states and molecular pathways. These limitations underscore the need for multi-center collaborative efforts to collect and analyze data from more cases of this rare tumor.

## Conclusion

This case highlights a rare presentation of MTT with primary thoracic involvement in a young patient with NF1. Despite the tumor’s aggressive biology and early postoperative recurrence, this case demonstrates that sustained disease control can be achieved through a personalized, multimodal approach combining immunotherapy, targeted therapies, and chemotherapy.

## Data Availability

The data is deposited in the CNSC database under the accession number CNP0009193, and the corresponding data link is available at: https://db.cngb.org/cnsa/project/CNP0009193_286f8dab/reviewlink/.

## Supplementary material

Supplementary Figure 1 scRNA-seq profiling of the MTT reveals a hyper-proliferative malignant clone with metabolic plasticity and EMT features within an immunosuppressive microenvironment.

## References

[1] Al-Alwan M Noar GA Al-Alwan A Alrwabdeh S Etawi M . Sporadic Malignant triton tumor of shoulder: a case report. Med Arch. (2024) 78:174–6. doi: 10.5455/medarh.2024.78.174-176, PMID: 38566863 PMC10983085

[2] Vilanilam GK Nayar D Pandey I Vattoth S . Recurrent sporadic Malignant triton tumor in the carotid sheath in the absence of neurofibromatosis. Neuroradiol J. (2024) 37:376–80. doi: 10.1177/19714009231196476, PMID: 37608426 PMC11138328

[3] AlAli BM Amr SS . Malignant glandular triton tumor arising in the radial nerve with prolonged survival: A case report and review of the literature. Case Rep Pathol. (2021) 2021:4614185. doi: 10.1155/2021/4614185, PMID: 33791136 PMC7997754

[4] de Traux de Wardin H Dermawan JK Vanoli F Jiang SC Singer S Chi P . NF1-driven rhabdomyosarcoma phenotypes: A comparative clinical and molecular study of NF1-mutant rhabdomyosarcoma and NF1-associated Malignant triton tumor. JCO Precis Oncol. (2024) 8:e2300597. doi: 10.1200/PO.23.00597, PMID: 38603649 PMC11161258

[5] Hashimoto K Nishimura S Goto K . Advanced metastatic Malignant triton tumor in neurofibromatosis type 1: A case report and management challenges. Cancer Diagn Progn. (2025) 5:410–6. doi: 10.21873/cdp.10454, PMID: 40322210 PMC12046655

[6] Ban Y Oebisu N Yao H Takada N Hoshi M Nakamura H . Malignant triton tumor of the distal femur: A case report and review of the literature. In Vivo. (2024) 38:3112–6. doi: 10.21873/invivo.13796, PMID: 39477416 PMC11535929

[7] Barh A Mukherjee B Koka K Krishnakumar S . Triton tumor of the orbit. Orbit. (2020) 39:418–21. doi: 10.1080/01676830.2019.1692873, PMID: 31746248

[8] Catelas DN Pita S Coelho A Oliveira V Cardoso P . Malignant “triton” tumor of the lower extremity with a history of fracture. Rev Esp Patol. (2024) 57:59–63. doi: 10.1016/j.patol.2023.06.004, PMID: 38246712

[9] Fader AN Roque DM Siegel E Buza N Hui P Abdelghany O . Randomized phase II trial of carboplatin-paclitaxel compared with carboplatin-paclitaxel-trastuzumab in advanced (Stage III-IV) or recurrent uterine serous carcinomas that overexpress her2/neu (NCT01367002): updated overall survival analysis. Clin Cancer Res. (2020) 26:3928–35. doi: 10.1158/1078-0432.CCR-20-0953, PMID: 32601075 PMC8792803

[10] Seddighzadeh RP Brower S Tzeng J Serur A . Malignant triton tumor below the peritoneal reflection: a case report. J Surg Case Rep. (2020) 2020:rjaa171. doi: 10.1093/jscr/rjaa171, PMID: 32595924 PMC7303023

[11] Ghafouri RS Hakim NM Konstantinidis IT Kafchinski L Philipovskiy A . A rare case of progressive Malignant triton tumor with rare somatic mutation in TSC2 gene. Anticancer Res. (2021) 41:3029–36. doi: 10.21873/anticanres.15085, PMID: 34083294

[12] Shah H Tailor P Shah K Shah T . A rare case of Malignant triton tumor without associated neurofibromatosis. Cureus. (2024) 16:e69016. doi: 10.7759/cureus.69016, PMID: 39385882 PMC11463885

[13] Hou Z Wang C Li L Dong L . Retroperitoneal Malignant triton tumor in an infant: a case report and literature review. Transl Pediatr. (2020) 9:567–72. doi: 10.21037/tp.2020.03.12, PMID: 32953555 PMC7475308

[14] Jiang TL Liu Y Ji B Sheng DH He QC Song JC . Malignant triton tumor of uterus: A case report and literature review. J Clin Ultrasound. (2024) 52:331–7. doi: 10.1002/jcu.23630, PMID: 38126255

[15] Kumar KP Kohli P Kumar SD Jagadesan P Penumadu P . ‘Triton’ Tumor of the lower alveolus: an aggressive variant of Malignant peripheral nerve sheath tumour. Indian J Otolaryngol Head Neck Surg. (2022) 74:5861–4. doi: 10.1007/s12070-021-02473-4, PMID: 36742747 PMC9895465

[16] Rekhi B Jambhekar NA Puri A Agrawal M Chinoy RF . Clinicomorphologic features of a series of 10 cases of Malignant triton tumors diagnosed over 10 years at a tertiary cancer hospital in Mumbai, India. Ann Diagn Pathol. (2008) 12:90–7. doi: 10.1016/j.anndiagpath.2007.04.010, PMID: 18325468

[17] Daimaru Y Hashimoto H Enjoji M . Malignant “triton” tumors: a clinicopathologic and immunohistochemical study of nine cases. Hum Pathol. (1984) 15:768–78. doi: 10.1016/S0046-8177(84)80169-0, PMID: 6235165

[18] Tsimpinos M Pigadiotis E Kontaxis V Lioulias A . Giant Malignant Triton tumour of the posterior mediastinum. Interact Cardiovasc Thorac Surg. (2021) 33:657–9. doi: 10.1093/icvts/ivab142, PMID: 34041530 PMC8923424

[19] Melnichenko I Sargsyan L Bedirian K Dallakyan D Gevorgyan A Aloyan G . How to treat metastatic Malignant triton tumor in an adolescent. Oncol (Williston Park). (2022) 36:674–7. doi: 10.46883/2022.25920979, PMID: 36445979

