# Supplementary figures and images for: Malignant triton tumor with thoracic region as the initial presentation: a case report
Source: Front Oncol. 2026 Mar 27;16:1764543. doi: 10.3389/fonc.2026.1764543 (PMC13065672; doi:10.3389/fonc.2026.1764543)

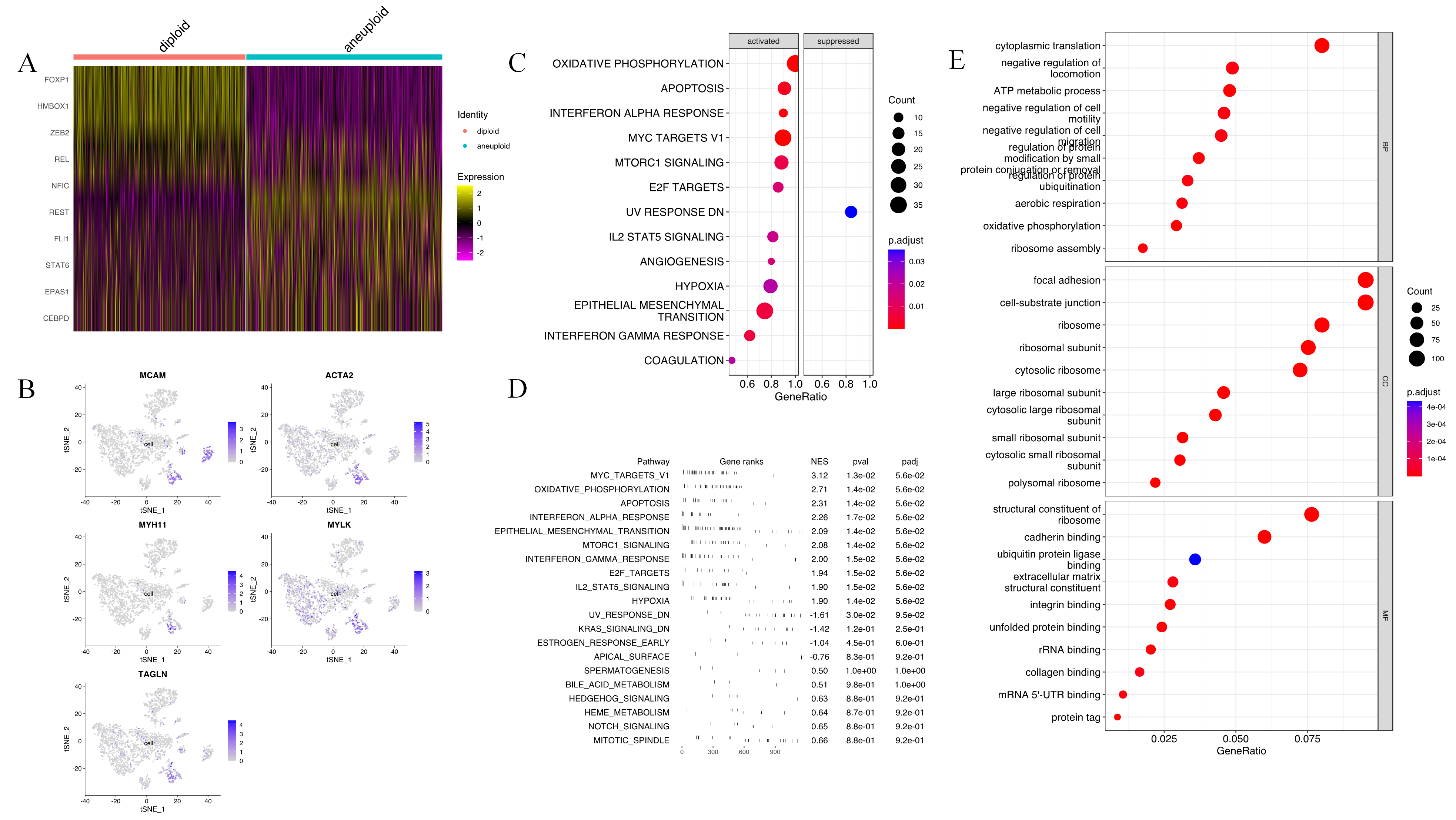

Supplement: Supplementary Figure 1 — scRNA-seq profiling of the MTT reveals a hyper-proliferative malignant clone with metabolic plasticity and EMT features within an immunosuppressive microenvironment. [file Image1.tif]
